# YSQ‐GeMS: Development of a Short Form of the Young Schema Questionnaire for Geriatric Mental Health Care Using Item Response Theory

**DOI:** 10.1002/cpp.70233

**Published:** 2026-02-16

**Authors:** L. Botter, P. F. M. Krabbe, D. L. Gerritsen, S. D. M. van Dijk, R. C. Oude Voshaar

**Affiliations:** ^1^ Department of Psychiatry University of Groningen, University Medical Center Groningen Groningen the Netherlands; ^2^ Atlant, Markenhaven, Center for Specialized Chronic Psychiatric Nursing Home Care University of Groningen Beekbergen the Netherlands; ^3^ Department of Epidemiology University of Groningen, University Medical Center Groningen Groningen the Netherlands; ^4^ Department of Primary and Community Care Radboud University Medical Center, Radboud Institute for Medical Innovation Nijmegen the Netherlands

**Keywords:** item response theory, long‐term care, nursing home care, older adults, schema therapy, young schema questionnaire

## Abstract

Schema therapy is effective for older adults with personality disorders, but the Young Schema Questionnaire (YSQ) may be too lengthy for use in geriatric mental health care. This study evaluated the construct validity of the YSQ‐L2 in adults aged 35–97 years receiving residential or outpatient geriatric, psychiatric or medical care and developed a shorter form for this population. Data from 214 participants from Dutch nursing homes and outpatient services were analysed using Item Response Theory across 16 YSQ‐L2 subscales. Each subscale was reduced to five items based on discrimination, difficulty, Differential Item Functioning for cognitive status and face validity. Most items showed adequate discrimination and coverage, whereas the Enmeshment scale had limited sensitivity and the Social Undesirability scale was excluded. The resulting 75‐item YSQ‐GeMS showed strong concordance with the original, providing a psychometrically robust and efficient tool for schema assessment in geriatric mental health care.

## Summary

- The YSQ‐GeMS is a psychometrically robust, substantially shorter alternative to the original YSQ−L2, with strong concordance and reduced respondent burden.
- Items susceptible to cognitive impairments were excluded, improving suitability for cognitively vulnerable older adults.
- Brief schema assessment may support wider implementation of schema therapy in nursing homes and other geriatric care settings, enabling more individualized interventions.

## 1 Introduction

Personality disorders are notably prevalent among nursing home residents, posing significant challenges for care delivery. Prevalence rates vary from 13% in general nursing home units to 24% (even 44% when clinically observed personality disorders are included) in specialized gerontopsychiatric units (Collet et al. 2018; Van Den Brink et al. 2017). This is likely accounted for by the finding that older adults with personality disorders are at a higher risk for nursing home admission (Miller and Rosenheck 2006). Individuals with personality disorders experience a high disease burden and long term care professionals encounter significant challenges in managing the interpersonal difficulties that frequently arise (Himelick and Walsh 2002; Soeteman et al. 2008). Accumulating evidence supports the effectiveness of schema therapy in treating personality disorders, as well as chronic mood and anxiety disorders, leading to its widespread adoption (Jacob and Arntz 2013; Körük and Özabaci 2018; Peeters et al. 2022; Van Dijk et al. 2023). Despite its limited application in older adults, a recent randomized controlled trial demonstrated that schema group therapy is also effective in this population. The study found moderate effect sizes for improvements in affective symptoms and a small effect for the improvement in personality functioning (Veenstra‐Spruit et al. 2024). Furthermore, two case reports provide preliminary results for the effective application of schema therapy in nursing home residents without dementia (Botter et al. 2022; Lapp et al. 2019). This warrants a shift from therapeutic nihilism (De Leo et al. 1999; Hunt 2007) to psychotherapeutic optimism for older adults with personality disorders. However, the proper delivery of schema therapy currently requires the use of lengthy and complex self‐report questionnaires for pre‐treatment assessment and outcome monitoring, predominantly relying on the 205‐item Young Schema Questionnaire (YSQ) (Young 1990). Although a third, shortened version of the YSQ has been introduced (Young 2014), a preliminary psychometric evaluation in 104 community‐dwelling older adults demonstrated low internal consistency for five of the 18 schemas (Phillips et al. 2019). This may imply that item reduction was suboptimal for older adults. Furthermore, the availability of a brief YSQ with comparable psychometric properties across geriatric and institutionalized populations would facilitate broader clinical implementation of schema therapy in nursing homes and other residential care settings.

Schema therapy targets early maladaptive schemas: dysfunctional and emotionally charged core beliefs that a person can have about the self, others and the world. Early maladaptive schemas are formed at a young age when basic emotional needs are not met by the significant caretakers of the child (Young et al. 2003). Persistence of early maladaptive schemas into old age is likely, due to the self‐perpetuating nature of early maladaptive schemas (Schmidt et al. 1995). Early maladaptive schemas can be identified by the Young Schema Questionnaire—Long Form (YSQ‐L2) (Young and Brown 1994). The YSQ‐L2 is a self‐report instrument that measures the presence of 16 early maladaptive schemas, which were originally theorized by Young (1990). Construct validity has been examined by several studies that confirmed the 16‐factor structure of the YSQ‐L2 with support of good internal consistency (Pauwels et al. 2013; Rijkeboer and Van Den Bergh 2006).

To identify the most appropriate items for assessing maladaptive schemas in a nursing home population, item selection should be based on analyses starting from the original 205‐item, comprehensive version of the instrument, i.e., the YSQ‐L2. However, the YSQ‐L2 has not yet been studied in nursing home populations and only limited research exists in older adults. Pauwels et al. (2014) examined age‐neutrality using differential item functioning (DIF) in a sample with alcohol use disorders. The findings showed that 97% of items exhibited no DIF, suggesting the response on the majority of items was not influenced by the age of the respondent. However, their ‘older adult’ group had a low mean age (63.3, SD = 3.3), likely including few participants over 70 and none over 75. Many respondents may still have been employed, limiting sensitivity to item bias relevant to older or residential care populations, particularly for items involving occupational or social functioning. Moreover, the small and demographically narrow subgroup (*n* = 107) may have lacked power to detect subtle effects.

Considering the lack of psychometric evaluation in the oldest‐old, residential care settings and cognitively impaired populations, we aimed to examine which items of each subscale are most informative and valid when used in a geriatric mental health care setting and whether each schema can be reliably and validly measured by only five items in this population. This will result in the development of a brief YSQ‐GeMS (Young Schema Questionnaire for Geriatric Mental health care Short form), designed for efficient schema assessment in older and institutionalized populations.

## 2 Methods

### 2.1 Participants

A total of 214 participants with a psychiatric or geriatric care need were recruited in Dutch NHs and outpatient geriatric mental health facilities. Participants were recruited across different care settings to ensure item calibration across the entire latent trait continuum relevant to geriatric mental health care. The sample is comprised of 61.7% women and 38.3% men with a mean age of 69. Of these participants, 25.9% had a lower education, 37.3% completed a form of middle‐level education and 36.8% had a higher level of education. All participants possessed legal capacity regarding participation in the present study. Participants were excluded when there was an active episode of a severe mental disorder such as an active bipolar disorder, psychotic disorder or active substance use disorders, but also severe depressive episodes. Exclusion furthermore followed when candidates were diagnosed with current delirium, any type of (clinically) suspected dementia or extensive acquired brain damage accompanied by severe communicative problems or impairments of the sense of self thus impeding the ability to self‐report. Exclusion criteria were judged based on inspection of the medical files and clinical judgement on the current mental state of participants by the treating clinicians.

### 2.2 Procedures

Inclusion and exclusion criteria were checked by the department staff, after which oral and written study information was given to all eligible patients by the researcher. After having provided written informed consent, participants were requested to fill in self‐report questionnaires and received an appointment for the neuropsychological testing. Assistance with the questionnaires was provided if participants found the self‐report questionnaires too strenuous. In such cases, items were read aloud and the Likert scale response options were displayed visually to facilitate completion. Additional informed consent was requested from 60 participants (15 of each group), for a second smaller assessment within 2 weeks to examine the test–retest reliability.

This study was approved by an institutional ethics committee and all procedures were conducted in accordance with the Declaration of Helsinki.

### 2.3 Measures

#### 2.3.1 YSQ‐L2

The YSQ‐L2 (long form, 2nd version) is a self‐report questionnaire consisting of 205 items. Items are rated on a 6‐point Likert rating scale ranging from 0 ‘completely untrue for me’ to 6 ‘describes me perfectly’. The YSQ‐L2 assesses 16 different early maladaptive schemas: Emotional Deprivation (9 items), Abandonment/Instability (18 items), Mistrust/Abuse (17 items), Social Isolation (10 items), Defectiveness/Shame (15 items), Failure to Achieve (9 items), Dependence/Incompetency (15 items), Vulnerability to Harm/Illness (14 items), Enmeshment (11 items), Subjugation (10 items), Self‐Sacrifice (17 items), Emotional Inhibition (9 items), Unrelenting Standards (16 items), Entitlement (11 items), Insufficient Self‐Control (15 items) and Social Undesirability (9 items). The mean of the aggregated item scores within each subscale represents the degree of activation of a specific Early Maladaptive Schema. A higher score across a subscale reflects a stronger presence of that particular early maladaptive schema and thus more pathological personality functioning. The YSQ‐L2 does not yield a total score summarizing overall personality functioning across all early maladaptive schemas. The YSQ‐L2 has been translated in Dutch by Sterk and Rijkeboer (1997) in accordance with the standard practice, including back translation and was approved by Jeffrey Young, author of the original questionnaires. Two studies on the performance of the Dutch YSQ‐L2 in Dutch populations showed favourable reliability and validity (Rijkeboer et al. 2005; Rijkeboer and Van Den Bergh 2006).

#### 2.3.2 Cognitive Functioning

Cognitive impairments are common in older adults receiving mental health care and may be present in the absence of a neurodegenerative disorder. Therefore, the Montreal Cognitive Assessment (MoCA) was administered as a global measure of cognitive functioning. The MoCA is a brief pen‐and‐paper neuropsychological test, clinically used for screening purposes (Nasreddine 2005). Small assignments are used to test visuospatial and executive functioning, naming, attention, memory (direct and delayed recall of new information), language, abstraction and orientation. The sum score ranges from 0 to 30 with validated cut‐off score of 24 to detect cognitive dysfunction in older adults (Malek‐Ahmadi and Nikkhahmanesh 2024).

### 2.4 Statistical Analysis

Item Response Theory (IRT) is a psychometric measurement model used to analyse and scale the relationship between individuals' responses to items (questions) and their position on the underlying trait the items are intended to measure. IRT offers a robust framework for assessing the validity and reliability of patient‐reported outcome measures, assuming that the latent trait being measured is unidimensional. IRT addresses certain aspects of construct validity by examining which items of the YSQ‐L2 provide sufficiently reliable information and which sections of the construct are covered by the scale's items. While classical test theory (CTT) evaluates how well items relate to the total score, IRT models the distinct relationship between an individual item and the underlying latent trait. This enables more precise questionnaire refinement by identifying and removing redundant or low‐performing items without reducing measurement accuracy. A key advantage of IRT over CTT is that both items and response categories are weighted: items vary in their contribution based on discrimination and response categories are not assumed to be equally spaced (e.g., weighted as 0.6, 1.8, 3.6, 4.3, 5.2 and 5.8 instead of 1, 2, 3, 4, 5 and 6), resulting in more accurate scoring.

In the IRT analyses, missing values (i.e., individual item responses) on YSQ‐L2 variables were automatically omitted without listwise deletion of entire cases. Missing values per item ranged from 3.3% to 4.7%. Category thresholds were inspected to confirm appropriate ordering and item characteristic curves (ICCs) were visually examined to assess the monotonicity of response options. IRT analyses were conducted on the full sample without stratification by recruitment setting, to allow for item calibration across the entire latent trait distribution.

The Graded Response Model (GRM) was employed as the IRT model suitable for polytomous items. Item fit was evaluated based on two parameters: (1) discrimination (parameter *a*), which indicates how well an item differentiates between individuals at different levels of the latent trait and reflects the steepness of the item response curve; and (2) difficulty (parameter *b*), which reflects the severity of the underlying trait (i.e., schema) on the standardized theta (θ) scale. Item fit was also visualized through Boundary Characteristic Curves (BCCs) to examine how well response options are ordered and at which trait levels each option is most likely to be chosen. ICCs were subsequently produced, first per response category, then aggregated per item, to assess how well an item discriminates between individuals and where along the trait continuum the item is most informative. Items were then located on the theta scale (−3 to +3) to evaluate their distribution. CTT total scores (based on standard mean score computation of all items in a scale) were compared with IRT‐based scores using scatterplots to assess their relationship and histograms to inspect distributional patterns.

Subsequently, overlapping items on the theta scale were examined to shorten each subscale, preferably to five items. Items were grouped into five θ‐segments to ensure sufficient coverage across the latent trait continuum. Within each segment, items with the highest discrimination parameters were selected. No fixed cut‐offs were applied, as universally accepted thresholds for item discrimination are lacking. In cases where differences in item discrimination values were minimal (i.e., less than 0.5), authors (LB, SvD and RCOV), as experienced schema therapy clinicians, selected the most appropriate items based on face validity. Face validity was operationalized and assessed by three criteria: relevance to the construct, clarity of wording and brevity. The authors first rated the items independently, after which a meeting was held to reach consensus through discussion. The choice of the number of items per schema was guided by psychometric and practical considerations. In the next step, the newly constructed scales were again fitted using the same GRM procedure, assuring that items had retained sufficient discrimination levels and adequate distribution of their degree of difficulty. Scatterplots were visually examined to check for profound changes in correlation between total scores and IRT scores.

To evaluate whether items functioned differently in the presence of cognitive impairment, DIF analyses were conducted using ordinal logistic regression. For each item, uniform and non‐uniform DIF were tested separately using likelihood ratio tests. Uniform DIF indicates that an item is systematically easier or harder for one subgroup across all levels of the underlying trait, while non‐uniform DIF reflects an interaction between group membership and the latent trait level—i.e., the magnitude or direction of DIF varies depending on the trait level. Subgroups were defined by cognitive impairment defined as a MoCA score < 24 (yes/no). The Benjamini–Hochberg procedure was applied to account for multiple comparisons within each scale. Items were flagged for DIF when the adjusted *p*‐value fell below the 0.05 threshold and were precluded from item selection. All analyses were conducted using Stata, version 18.0 (BE; StataCorp, College Station, TX). The authors used OpenAI's ChatGPT (2024 version) to assist in language and table editing. The AI tool was not used for data analysis, content generation or interpretation of results. All content was reviewed and finalized by the authors.

## 3 Results

### 3.1 Study Sample

The final study sample consisted of 214 participants, with a mean age of 69 years (SD = 11; range = 35–97). Age significantly differed across the recruitment settings. Participants from somatic care units in nursing homes (mean = 82 years, SD = 9) and geriatric outpatients (mean = 73 years, SD = 7) were older than those from specialized gerontopsychiatric units (mean = 67 years, SD = 11) and outpatient psychiatry (mean = 63 years, SD = 9) (all *p*‐values < 0.001). The sample was comprised of 61.7% women and 38.3% men, with no statistically significant differences in sex distribution across the groups (*χ* ^2^, *p* = 0.247). Educational level, however, showed significant variation across settings (*χ* ^2^, *p* < 0.001). Lower educational levels were most prevalent in participants from specialized gerontopsychiatric units (47.1%) and somatic care units (40.7%). In contrast, higher education was more common among participants from the outpatient psychiatry (55.1%) and outpatient geriatric groups (38.5%). These differences in demographic characteristics across settings were not included as grouping variables in the IRT models, which were conducted on the full sample to support overall item selection and scale assessment. Participants displayed mild cognitive impairment on average, as reflected by a mean MoCA score of 23.0. The relatively wide standard deviation (5.0) further indicates notable variability in cognitive performance across individuals.

### 3.2 DIF

Out of the 205 items, 9 demonstrated significant DIF between individuals with and without cognitive impairment and were thus precluded from the abbreviated version. Uniform DIF was identified in six items, distributed across the schemas Abandonment/Instability, Social Undesirability (two items), Subjugation, Entitlement and Enmeshment. Non‐uniform DIF was found in four items, associated with the schemas Social Undesirability, Entitlement, Defectiveness/Shame and Enmeshment. One item within the Entitlement schema showed both uniform and non‐uniform DIF. In terms of direction, four of the nine items showed higher expected item scores among cognitively impaired individuals (suggesting potential overestimation of schema endorsement), while five items showed the opposite pattern. The exclusion of three Social Undesirability items due to DIF ultimately contributed to the removal of the entire subscale from the short form.

### 3.3 Item Response Theory Analysis: Abandonment/Instability Scale

In practice, many short form instruments retain approximately 4 to 6 items per construct to balance reliability, construct coverage and feasibility, although no fixed standard exists and optimal scale length depends on context and measurement goals. Options of 5, 6 and 7 items per schema were empirically evaluated; scatterplots comparing classical test theory‐based and IRT‐based scores showed only modest differences in information loss. Given comparable psychometric performance and lower respondent burden, the 5‐item solution was retained.

Considering the complexity of IRT analyses, we present the results of the Abandonment/Instability scale (18 items) of the YSQ‐L2 in detail as an example and refer to the supplemental material for the results on the other 15 subscales.

**FIGURE 1:**
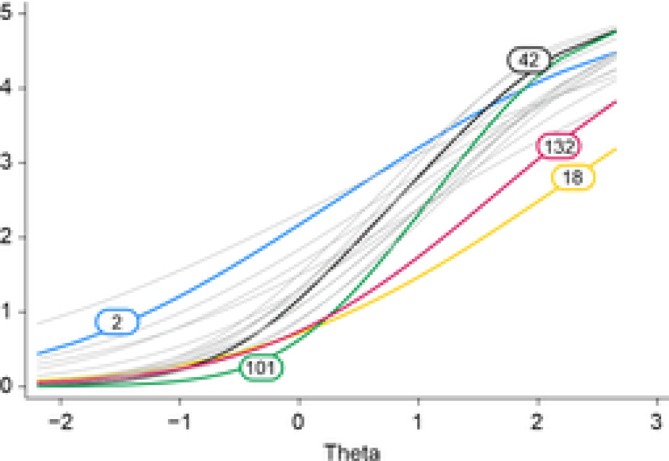
Item characteristic curves of the abandonment/instability‐scale. Curves reflecting the items selected for the 5‐item short form of the scale are highlighted.

**FIGURE 2:**
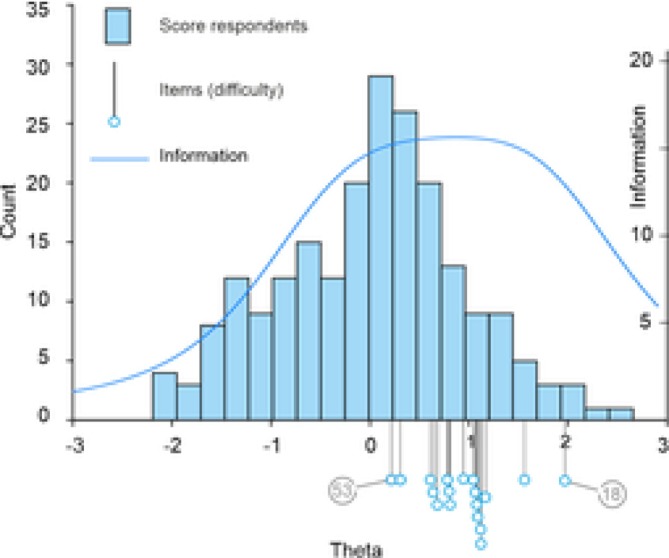
Visual overview of performance of the full length abandonment/instability scale, displaying across the theta (x‐axis): the test information function, the distribution of respondents' scores and item difficulty.

The 18 individual items of Abandonment/Instability scale were completed by 205 to 207 (variation due to item‐level analyses) of the 214 participants. Descriptive statistics per item showed predominant endorsement of low to middle response categories, with fewer counts of item responses distributed on the upper end of the Likert scale. Inspection of the BCCs demonstrated adequate ordering of response categories of the items. This implies that response categories were reliable in distinguishing respondents based on their level of Abandonment/Instability. However, when the slopes were aggregated to form ICCs (Figure 1), item distribution across the theta demonstrated to be limited to the higher range of the theta (between 0 and 2), with limited sensitivity in the lower range (from −2 to 0). Figure 2 provides a visual overview of the test information function, respondent score distribution and item difficulty across the theta continuum, illustrating that the scale yields the greatest information at moderate‐to‐high schema activation levels while showing limited sensitivity in the lower range.

**FIGURE 3:**
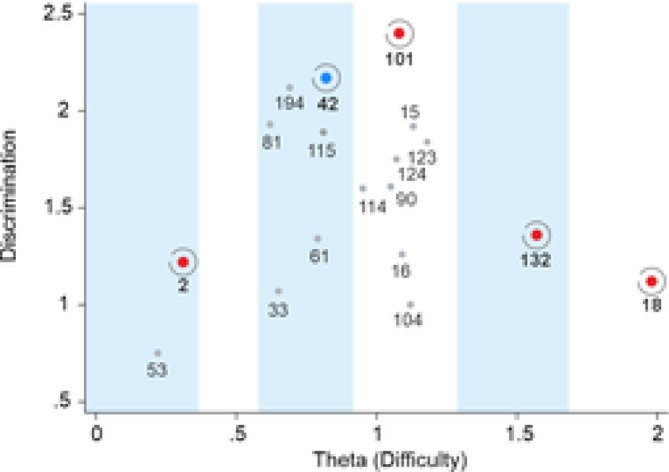
Distribution of item discrimination and selection for short form development. *Note:* Each dot represents an item plotted by its location on the theta scale (x‐axis) and discrimination parameter (y‐axis). Items were grouped into five pools for further selection, as reflected by blue and white columns. Red‐highlighted items were selected based primarily on IRT parameters; the blue‐highlighted item was selected based on face validity.

Discrimination parameters (a) varied between 0.75 and 2.40, indicating generally acceptable differentiation capacity. The difficulty parameters (b) showed a clustering towards higher theta values (range 0.22–1.98), implying some redundancy in measurement coverage at this range. The full set of discrimination and difficulty parameters for all items is summarized in Table 1. Visual inspection of adjacent item grouping was used to identify theta ranges with potential item redundancy. Within each range, the item providing the highest information value—based on its discrimination parameter—was selected to ensure adequate theta coverage (Figure 3). In one of the five identified theta segments, four items (42, 81, 115 and 132) showed similar discrimination values. Item 42 was ultimately selected based on face validity. The newly constructed 5‐item version of the subscale displayed a well‐balanced distribution of item difficulties and maintained strong discriminative properties (Table 2).

**TABLE 1:** Item response theory parameters based on the graded response model.

	Items																	
	2	15	16	18	33	42	53	61	81	90	101	104	114	115	123	124	132	194
Discrimination coefficient	1.22	1.92	1.26	1.12	1.07	2.17	0.75	1.34	1.93	1.61	2.40	1.00	1.60	1.89	1.84	1.75	1.36	2.12
Resp. category difficulty																		
> = 2	−1.31 (0.22)	−0.01 (0.11)	−0.33 (0.15)	0.38 (0.16)	−0.93 (0.21)	−0.16 (0.11)	−1.35 (0.34)	−0.98 (0.18)	−0.41 (0.13)	−0.12 (0.13)	0.23 (0.10)	−0.54 (0.19)	−0.31 (0.13)	−0.38 (0.12)	−0.08 (0.12)	−0.19 (0.12)	0.34 (0.14)	−0.42 (0.12)
> = 3	−0.53 (0.16)	0.63 (0.13)	0.40 (0.15)	1.38 (0.25)	−0.02 (0.16)	0.22 (0.11)	−0.59 (0.24)	0.05 (0.14)	0.11 (0.11)	0.51 (0.13)	0.68 (0.12)	0.14 (0.17)	0.42 (0.13)	0.27 (0.11)	0.66 (0.13)	0.52 (0.12)	1.11 (0.19)	0.31 (0.11)
> = 4	0.38 (0.15)	1.13 (0.16)	1.19 (0.20)	2.20 (0.37)	−0.16 (15.5)	0.84 (0.13)	0.20 (0.21)	0.95 (0.17)	0.60 (0.12)	1.25 (0.18)	1.14 (0.14)	1.29 (0.26)	0.93 (0.15)	0.80 (0.13)	1.26 (0.17)	1.06 (0.15)	1.68 (0.26)	0.83 (0.13)
> = 5	1.07 (0.19)	1.71 (0.22)	1.85 (0.28)	2.74 (0.47)	1.31 (0.23)	1.4 (0.17)	1.09 (0.29)	1.47 (0.22)	1.19 (0.16)	1.62 (0.22)	1.45 (0.17)	1.94 (0.34)	1.66 (0.22)	1.40 (0.18)	1.75 (0.22)	1.65 (0.21)	2.05 (0.31)	1.12 (0.15)
= 6	1.97 (0.29)	2.18 (0.28)	2.36 (0.35)	3.19 (0.56)	2.19 (0.35)	1.84 (0.21)	1.77 (0.40)	2.46 (0.35)	1.63 (0.20)	2.00 (4.9)	1.92 (0.22)	2.79 (0.48)	2.06 (0.27)	1.93 (0.23)	2.28 (0.29)	2.33 (0.30)	2.68 (0.42)	1.61 (0.19)
Mean difficulty	0.31	1.13	1.09	1.98	0.65	0.82	0.22	0.79	0.62	1.05	1.08	1.12	0.95	0.81	1.18	1.07	1.57	0.69

**TABLE 2:** Newly structured 5‐item scale abandonment/instability, with optimal difficulty distribution, selected based on the highest discrimination parameters.

Item	Difficulty (18 item GRM)	Difficulty (5 item GRM)	Discrimination (18 item GRM)	Discrimination (5 item GRM)
2	0.31	0.30	1.22	1.35
42	0.82	0.92	2.17	1.71
101	1.08	1.03	2.40	2.94
132	1.57	2.00	1.36	1.17
18	1.98	1.85	1.12	0.98

**FIGURE 4:**
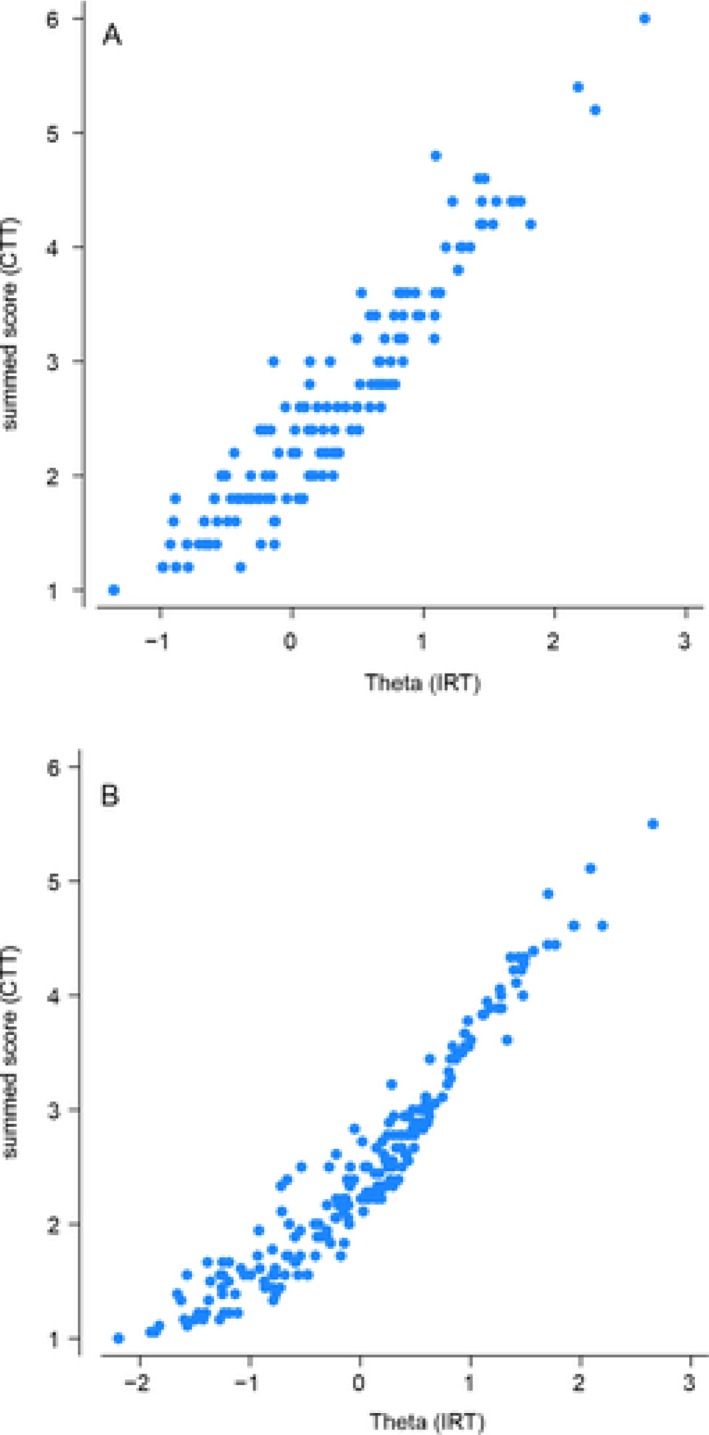
Scatterplots displaying standard vs. item response theory scores, for the short form (A) and full length scale (B).

Concurrent validity of the short form subscales was supported by scatterplot analyses (Figure 4), which demonstrated a high degree of concordance between IRT‐based scores and CTT total subscale scores. This relationship was strong for the full‐length version and, while slightly reduced, remained sufficiently robust for the short form version of the scale.

### 3.4 Summary of IRT Findings Across Other Scales

The IRT‐guided item selection process ultimately resulted in a shortened version of the YSQ‐L2 consisting of 75 items, with 15 subscales each reduced to five items. The remaining 15 scales (Table 3) exhibited theta ranges that varied from somewhat limited (e.g., Emotional Inhibition: 1.07–1.52) to considerably broad (e.g., Unrelenting Standards: −0.09–2.36). Most scales demonstrated comparable coverage of the latent trait continuum, with an average theta range of 1.41. Item discrimination values were generally adequate. Scale abbreviation to five items was initially performed by analysing IRT parameters—namely, theta distribution and discrimination power. Of the 75 selected items, 27 were chosen based on face validity, as their discrimination values were comparable to those of adjacent items within the same theta range.

**TABLE 3:** All 16 early maladaptive schemas of the YSQL2 and their item response theory parameters based on the graded response model, for the original and abbreviated 5‐item scales.

Early maladaptive schemas	*N* ^a^	Discrimination range		Theta range			
		18‐item scale	5‐item scale	18‐item scale	5‐item scale	Items with DIF (*p* < 0.05)	Selected items of the 5‐item scale
Emotional deprivation	9	1.25–2.86	1.27–2.94	0.16–1.51	0.75–1.60	—	44, 96^c^, 121^c^, 155, 184
Abandonment/instability	18	0.75–2.40	1.31–1.98	0.22–1.98	0.30–2.00	81	2, 18, 42^c^, 101, 132
Mistrust/abuse	17	0.85–2.52	1.32–2.68	0.05–1.75	0.04–1.71	—	11, 24, 60^c^, 127, 130^c^
Social isolation	10	0.80–3.18	1.61–2.92	0.56–1.59	0.60–1.57	—	7, 31, 64, 103, 120
Defectiveness/shame	15	0.90–2.83	1.15–3.59	0.98–2.23	1.11–2.49	5	9, 47^c^, 82, 89, 137^c^
Failure to achieve	9	1.01–2.84	0.97–2.87	0.59–1.49	0.61–1.54	—	3, 4, 54, 107, 166
Dependence/in‐competency	15	0.90–2.07	0.89–3.34	−0.25–2.07	0.33–2.40	—	49, 52^c^, 75^c^, 117, 204^c^
Vulnerability to harm/illness	14	0.72–1.98	0.96–1.93	0.59–2.35	0.66–2.27	—	71, 98^c^, 110^c^, 134^c^, 198^c^
Enmeshment^b^	11	0.99–2.50	0.93–2.98	1.17–2.50	1.16–2.57	86, 138	99^c^, 143^c^, 154, 183, 188
Subjugation	10	1.06–2.66	0.92–3.19	0.31–0.85	0.29–2.04	72	40, 83^c^, 113, 142^c^, 193
Self‐sacrifice	17	0.64–2.25	0.96–2.62	−0.54–1.47	−0.62‐1.32	—	34^c^, 68, 160^c^, 196^c^, 199
Emotional inhibition	9	1.29–2.21	1.12–2.80	1.07–1.52	1.07–1.42	—	6, 36, 165, 170, 190
Unrelenting standards	16	0.66–2.25	1.04–2 03	−0.09–2.36	0.09–1.47	—	55^c^, 65, 147^c^, 203, 205^c^
Entitlement	11	0.84–2.27	−0.12–2.19	−0.14–1.90	1.06–2.46	182	93^c^, 102, 105, 111, 191
Insufficient self‐control	15	0.64–2.20	1.22–1.90	0.64–2.61	0.59–1.61	—	46, 51, 73^c^, 85^c^, 92
Social undesirability	9	0.37–2.52	1.57–1.74	0.02–2.46	1.08–2.00	35, 37, 116	—

^a^ Number of items per subscale/early maladaptive schema.

^b^ Normality compromised by spike in low scorers. Also visible in theta distribution.

^c^ Item selected based on face validity when discrimination performance differences of adjacent items were not conclusive.

Enmeshment was the sole subscale to show problems in normality distribution. The items clustered into two groups at the lower and upper ends of the theta range. To ensure optimal coverage, we prioritized theta distribution in the selection process. Consequently, we did not apply face validity to choose between items with similar discrimination values (i.e., items 190 and 201), as maximizing item distribution across the theta was considered more important.

Of the nine items comprising the Social Undesirability subscale, three exhibited DIF and two showed relatively low discrimination. As only four items remained with acceptable psychometric properties, the subscale was excluded from the short form. A detailed overview of the results for each schema scale is provided in the supplementary materials.

## 4 Discussion

This study aimed to explore item‐level indicators of construct validity of the YSQ‐L2 in older and/or cognitively impaired adults and to develop a psychometrically robust short form suitable for clinical use. Findings showed that the full item set demonstrated good item functioning and the resulting 75‐item short form retained key psychometric characteristics for 15 schemata while substantially reducing administration burden.

We examined the construct validity of the YSQ‐L2 in an older population including the oldest‐old and cognitively impaired individuals (excluding dementia). The first key finding is that items generally performed well exhibiting satisfactory discrimination levels, implying that YSQ‐L2 items are succinctly comprehensible and interpretable for respondents within this population. It can therefore be concluded that the items produce meaningful psychometric information on early maladaptive schema activation in older individuals with complex multi‐morbidity and cognitive impairments. This is in line with the conclusion by Pauwels et al. (2014) regarding age neutrality of YSQ items. However, as discussed previously, their sample lacked representation of the oldest‐old and cognitively impaired individuals. Our findings extend these observations by demonstrating that items also perform well in geriatric mental health care populations with complex care demands and including the oldest‐old. In contrast with their results, however, we found no discernible lower performance of items within the subscale Entitlement. If, as Pauwels et al. suggested, certain YSQ Entitlement items confound with impulsivity and impulsivity tends to decline with age, this effect may have been counteracted in our sample, considering that complex geriatric psychiatric populations frequently present with dysexecutive symptoms and thus continue to exhibit impulsivity into old age (Van Den Brink et al. 2017). Moreover, participants from a sample with addiction problems are likely to be more impulsive (Walther et al. 2012). This may have attenuated age‐related declines in impulsivity, making it less plausible that observed DIF can be attributed solely to age. Furthermore, unlike Pauwels et al. (2014), our analyses considered item‐specific IRT parameters, allowing us to detect item performance differences with greater precision. While Pauwels et al. relied primarily on DIF to assess age neutrality, they identified approximately 3% of items as DIF‐flagged. Our study, despite involving a more cognitively vulnerable population, yielded a comparable percentage of items (4%) showing DIF. This suggests that item functioning in the YSQ‐L2 is largely robust across aging and cognitive status, with only minor variation in item‐level measurement invariance.

Another important result of this study was that the range and distribution of theta values was somewhat limited to the higher end of the scales (0 to 2), implying that the instrument was especially sensitive in differentiating respondents that possess moderate to high activation levels of an early maladaptive schema. However, the range of theta values required to validly measure latent traits is always related to the aim and target population of the instrument (DeMars 2010). In clinical applications of the YSQ‐L2 among complex gerontopsychiatric populations with prevalent personality pathology, the range and distribution of theta values appears sufficiently sensitive to identify early maladaptive schemas, particularly considering the strong discriminative properties of the items. These findings support the use of the YSQ‐L2 as a valid instrument for schema assessment in older adults with psychiatric and cognitive difficulties. Nevertheless, the somewhat limited range of theta values observed in our study remains difficult to interpret, as IRT‐based evaluations of the YSQ‐L2 in general or adult populations are currently unavailable.

A final notable result was the development of a short form of the YSQ‐L2, to perform schema assessment efficiently in complex geriatric populations. All subscales exhibited redundancy of items on the theta, enabling reduction to five items per subscale. Short form items were selected through a combination of IRT parameters and face validity, ensuring that both statistical and clinical relevance guided item inclusion. Items that displayed DIF were excluded from the selection to ensure item endorsement is not influenced by cognitive dysfunction. The Social Undesirability subscale was excluded from the short form due to multiple psychometric limitations, including relatively low discrimination and several items exhibiting DIF. This decision is in line with previous findings indicating inconsistent performance of this subscale across studies. Notably, the schema has been removed altogether in a later version of the YSQ (Young et al. 2014). These developments suggest that Social Undesirability may lack distinct conceptual and psychometric utility, particularly in older or clinical populations. This resulted in a new short form of the YSQ comprised of 75 items, designed to decrease burdensome administration time for a vulnerable population, while preserving the psychometric quality of the YSQ. Based on our experience during data collection, administration of the full YSQ‐L2 typically took approximately 75 min. Given the 63% reduction in item count, the short form is expected to reduce completion time to around 28 min. In our transparent and reproducible selection procedure, we were able to preserve the range and distribution of theta values while retaining items with good discrimination properties. Concurrent validity of the short form was sufficient when IRT scores were compared with standard scores. Furthermore, our results indicate that the distribution of theta values of the Enmeshment scale is suboptimal in both the original 11‐item version and its shortened counterpart. This may be attributable to the fact that Enmeshment items often refer to the relationship with one's parents, which may be less relevant in older adults whose parents are usually no longer alive. Cautious interpretation of clinical results pertaining to this specific schema is therefore warranted in older populations characterized by mental–physical multimorbidity.

Although IRT‐based reduction allows for maintaining high measurement precision with fewer items, especially when selecting items with strong discrimination across the latent trait range, it does not prevent potential loss of content coverage. Kruyen et al. (2013) argue that shortening instruments may compromise construct validity, especially when driven solely by statistical criteria. In contrast, Embretson and Reise (Embretson and Reise 2000) emphasized that IRT facilitates informed test reduction by selecting items that preserve measurement quality. Therefore, our combined use of IRT‐based metrics and clinical content evaluation supports an optimized short form that balances psychometric precision with conceptual comprehensiveness. The development of the YSQ‐GeMS also holds practical relevance for increasing access to psychotherapy among older adults with personality pathology. By reducing assessment burden while preserving measurement quality, the short form enables more feasible implementation of schema therapeutic interventions in cognitively vulnerable geriatric populations, including nursing home residents. This contributes to expanding treatment opportunities beyond traditional supportive care and supports the integration of evidence‐based psychotherapeutic approaches in complex geriatric mental health settings.

A third and shortened version of the YSQ has been released (Young 2014) and validated for use by older, community‐dwelling adults (Phillips et al. 2019). The Dutch translation of the YSQ‐S3 was not yet published at the start of our study and was therefore not used to examine and abbreviate. Phillips et al. (2019) evaluated validity aspects of the YSQ‐S3 in a non‐clinical sample of 104 older adults aged 60–84. Although their findings generally supported reliability and validity, the limited sample size and use of CTT limit generalizability to more complex populations in which the YSQ is typically applied, such as individuals with personality pathology. Our IRT‐based approach allowed for greater precision in evaluating item performance across the latent trait continuum, facilitating the selection of items that offer complementary information and thus broad measurement of the trait. Moreover, two YSQ‐S3 items—found in the Enmeshment and Subjugation subscales—showed DIF in our sample based on cognitive status. Although the number is small, the absence of DIF‐screening in the YSQ‐S3 development process may limit its applicability in cognitively vulnerable populations. The YSQ‐GeMS explicitly excluded such items, enhancing its robustness for use in geriatric mental health care. Nonetheless, direct comparison between the YSQ‐S3 and the YSQ‐GeMS would be informative, as both assess the same late‐life schema constructs using different item selections and psychometric approaches. As the YSQ‐GeMS was derived from the YSQ‐L2 item pool future studies could combine YSQ‐L2 and YSQ‐S3 data from multiple older adult samples and apply IRT to enable item‐level linking across instrument versions despite differences in scale structure. Taken together, the findings of Phillips et al. (2019) and the current study strengthen the case for clinical use of schema assessment in older adults across varying levels of functioning and care settings.

Some methodological issues should be mentioned. For one, although our IRT analyses did not incorporate subgroup comparisons, it is important to acknowledge the significant differences in age and educational level across recruitment settings. Participants from somatic care units were notably older, while those from psychiatric outpatient settings were generally younger and more highly educated. These demographic variations may have influenced response styles or overall schema activation. Given that early maladaptive schemas are particularly prevalent in individuals with personality disorders, the absence of diagnostic information on personality pathology may be considered a limitation. This may partly account for the relatively modest levels of schema activation observed. Nonetheless, considering the predominantly psychiatric nature of both recruitment settings, personality pathology is likely to be highly prevalent, as studies report rates up to 33% among older psychiatric outpatients and up to 80% among inpatients (Oude Voshaar and Van Dijk 2025). Thus, future studies may need to investigate measurement invariance across age, educational attainment and presence of a personality disorder to determine whether items retain stable properties across diverse geriatric subpopulations. Another critical note is that we did not conduct confirmatory factor analysis (CFA) or examine convergent validity through comparison with established external instruments. While the present IRT‐based analyses offer valuable insights into item‐level psychometric functioning, further research is needed to more comprehensively evaluate the construct validity of the YSQ‐L2. This includes replication of findings in other clinical and non‐clinical older adult populations and the application of additional validation techniques such as CFA.

In conclusion, the proposed short form of the YSQ‐L2, the YSQ‐GeMS, offers a promising and efficient means of assessing early maladaptive schemas in geriatric mental health care, thereby facilitating access to psychotherapeutic interventions. Looking ahead, the development of computer‐adaptive testing—where item administration is tailored to individual response patterns—offers a direction for future refinement, combining precision with minimal respondent burden.

## Funding

Stichting Vermogensbeheer Hoogeland Zorg.

## Conflicts of Interest

The authors declare no conflicts of interest.

## Supporting information


**Table S1:** Item parameters for the Emotional Deprivation schema.
**Table S2:** Item parameters for the Mistrust/Abuse schema.
**Table S3:** Item parameters for the Social Isolation schema.
**Table S4:** Item parameters for the Defectiveness/Shame schema.
**Table S5:** Item parameters for the Social Undesirability schema.
**Table S6:** Item parameters for the Failure To Achieve schema.
**Table S7:** Item parameters for the Dependency/Incompetency schema.
**Table S8:** Item parameters for the Vulnerability To Harm schema.
**Table S9:** Item parameters for the Enmeshment schema.
**Table S10:** Item parameters for the Subjugation schema.
**Table S11:** Item parameters for the Self‐sacrifice schema.
**Table S12:** Item parameters for the Emotional Inhibition schema.
**Table S13:** Item parameters for the Unrelenting Standards schema.
**Table S14:** Item parameters for the Entitlement schema.
**Table S15:** Item parameters for the Insufficient Self‐control schema.
**Table S16:** Overview of YSQ‐GeMS items in Dutch, showing the preserved original item order, newly assigned item numbers and corresponding item numbers from the original YSQ‐L2.

## Data Availability

Due to legal and ethical constraints concerning participant privacy and the vulnerability of the study population, the data and materials are not publicly available. De‐identified data and analysis code may be made available upon reasonable request to the corresponding author, subject to ethical approval and data sharing agreements.
