# Supplementary material for: YSQ‐GeMS: Development of a Short Form of the Young Schema Questionnaire for Geriatric Mental Health Care Using Item Response Theory
Source: Clin Psychol Psychother. 2026 Feb 16;33(1):e70233. doi: 10.1002/cpp.70233 (PMC12908208; doi:10.1002/cpp.70233)
Supplement: Supplementary file 1 — Table S1: Item parameters for the Emotional Deprivation schema. Table S2: Item parameters for the Mistrust/Abuse schema. Table S3: Item parameters for the Social Isolation schema. Table S4: Item parameters for the Defectiveness/Shame schema. Table S5: Item parameters for the Social Undesirability schema. Table S6: Item parameters for the Failure To Achieve schema. Table S7: Item parameters for the Dependency/Incompetency schema. Table S8: Item parameters for the Vulnerability To Harm schema. Table S9: Item parameters for the Enmeshment schema. Table S10: Item parameters for the Subjugation schema. Table S11: Item parameters for the Self‐sacrifice schema. Table S12: Item parameters for the Emotional Inhibition schema. Table S13: Item parameters for the Unrelenting Standards schema. Table S14: Item parameters for the Entitlement schema. Table S15: Item parameters for the Insufficient Self‐control schema. Table S16: Overview of YSQ‐GeMS items in Dutch, showing the preserved original item order, newly assigned item numbers and corresponding item numbers from the original YSQ‐L2. [file CPP-33-e70233-s001.docx]

**Supplementary materials**
For: “YSQ-GeMS: Development of a Short Form of the Young Schema Questionnaire for Geriatric Mental Health Care Using Item Response Theory”

The following 15 tables present item-level discrimination and difficulty parameters for all items of the 15 schema subscales included in the Young Schema Questionnaire – Long Form, Second Version (YSQ-L2). Parameters for the *Abandonment/Instability* schema are reported in the main article. Each table corresponds to one schema and includes both the original full-scale items and those retained in the short form. For each item, item numbers and IRT discrimination and difficulty parameters are provided. A dash (-) indicates that an item was not included in the short form. For the *Social Undesirability* schema, no short form was created; the relevant columns are marked as Not Applicable (NA).

Finally, Table S16 presents the 75 items of the YSQ-GeMS in their new order, along with their corresponding original YSQ-L2 item numbers. The item content is provided in Dutch, as this version is publicly available open access, unlike the original English version.

| Emotional Deprivation | Original subscale | | 5-item subscale | |
| --- | --- | --- | --- | --- |
| Item | Discrimination | Difficulty | Discrimination | Difficulty |
| 44 | 1.37 | 1.51 | 1.27 | 1.60 |
| 77 | 1.81 | 0.81 | - | - |
| 96 | 2.26 | 0.96 | 1.60 | 1.14 |
| 97 | 2.18 | 0.66 | - | - |
| 121 | 2.05 | 0.61 | 2.94 | 0.57 |
| 155 | 1.25 | 1.25 | 1.59 | 1.07 |
| 177 | 2.11 | 0.78 | - | - |
| 184 | 2.86 | 0.74 | 2.05 | 0.81 |
| 192 | 2.48 | 0.96 | - | - |

Table S1. Item parameters for the Emotional Deprivation schema

Table S2. Item parameters for the Mistrust/Abuse schema

| Mistrust/Abuse | Original subscale | | 5-item subscale | |
| --- | --- | --- | --- | --- |
| Item | Discrimination | Difficulty | Discrimination | Difficulty |
| 8 | 1.69 | 1.09 | - | - |
| 11 | 2.15 | 1.20 | 2.23 | 1.21 |
| 20 | 2.52 | 0.93 | - | - |
| 23 | 1.46 | 1.12 | - | - |
| 24 | 1.35 | 1.75 | 1.43 | 1.71 |
| 29 | 1.65 | 0.87 | - | - |
| 58 | 1.47 | 0.64 | - | - |
| 60 | 2.06 | 0.72 | 1.97 | 0.72 |
| 69 | 1.89 | 0.80 | - | - |
| 127 | 1.05 | 0.05 | 1.32 | 0.04 |
| 130 | 2.43 | 1.04 | 2.68 | 1.01 |
| 150 | 2.16 | 0.52 | - | - |
| 164 | 1.94 | 0.96 | - | - |
| 169 | 0.85 | 1.46 | - | - |
| 172 | 1.84 | 0.66 | - | - |
| 176 | 1.81 | 1.34 | - | - |
| 181 | 1.97 | 0.79 | - | - |

Table S3. Item parameters for the Social Isolation schema

| Social Isolation | Original subscale | | 5-item subscale | |
| --- | --- | --- | --- | --- |
| Item | Discrimination | Difficulty | Discrimination | Difficulty |
| 7 | 2.64 | 0.91 | 2.78 | 0.91 |
| 31 | 2.18 | 0.87 | 2.04 | 0.89 |
| 64 | 3.18 | 0.70 | 2.92 | 0.73 |
| 66 | 2.82 | 0.66 | - | - |
| 70 | 1.94 | 0.64 | - | - |
| 80 | 1.17 | 0.65 | - | - |
| 103 | 2.72 | 0.58 | 2.49 | 0.60 |
| 120 | 1.55 | 1.59 | 1.61 | 1.57 |
| 128 | 1.83 | 0.56 | - | - |
| 173 | 0.80 | 0.66 | - | - |

Table S4. Item parameters for the Defectiveness/Shame schema

| Defectiveness/Shame | Original subscale | | 5-item subscale | |
| --- | --- | --- | --- | --- |
| Item | Discrimination | Difficulty | Discrimination | Difficulty |
| 1 | 1.93 | 1.13 | - | - |
| 5 | 1.59 | 1.76 | - | - |
| 9 | 1.34 | 2.23 | 1.15 | 3.71 |
| 12 | 1.57 | 1.36 | - | - |
| 19 | 1.31 | 0.98 | - | - |
| 41 | 1.97 | 1.25 | - | - |
| 43 | 1.31 | 1.80 | - | - |
| 45 | 1.89 | 1.11 | - | - |
| 47 | 2.87 | 1.29 | 3.25 | 1.25 |
| 74 | 2.61 | 1.29 | - | - |
| 82 | 2.78 | 1.02 | 2.18 | 1.11 |
| 87 | 1.91 | 1.25 | - | - |
| 89 | 2.99 | 1.46 | 3.59 | 1.42 |
| 137 | 1.52 | 1.72 | 1.48 | 1.75 |
| 148 | 2.06 | 1.16 | - | - |

Table S5. Item parameters for the Social Undesirability schema

| Social Undesirability | Original subscale | | 5-item subscale | |
| --- | --- | --- | --- | --- |
| Item | Discrimination | Difficulty | Discrimination | Difficulty |
| 14 |  | 1.29 | NA | NA |
| 28 |  | 0.92 | NA | NA |
| 35 |  | 1.24 | NA | NA |
| 37 |  | 1.06 | NA | NA |
| 57 |  | 0.02 | NA | NA |
| 116 |  | 2.46 | NA | NA |
| 145 |  | 2.23 | NA | NA |
| 163 |  | 1.88 | NA | NA |
| 175 |  | 1.77 | NA | NA |

Table S6. Item parameters for the Failure To Achieve schema

| Failure To Achieve | Original subscale | | 5-item subscale | |
| --- | --- | --- | --- | --- |
| Item | Discrimination | Difficulty | Discrimination | Difficulty |
| 3 | 1.69 | 1.39 | 1.97 | 1.31 |
| 4 | 1.01 | 1.49 | 0.97 | 1.54 |
| 38 | 1.26 | 1.34 | - | - |
| 54 | 1.80 | 1.18 | 1.36 | 1.38 |
| 95 | 2.03 | 1.02 | - | - |
| 106 | 2.12 | 0.62 | - | - |
| 107 | 2.84 | 0.59 | 2.64 | 0.61 |
| 158 | 2.01 | 1.06 | - | - |
| 166 | 2.56 | 1.07 | 2.87 | 1.04 |

Table S7. Item parameters for the Dependency/Incompetency schema

| Dependency/Incompetency | Original subscale | | 5-item subscale | |
| --- | --- | --- | --- | --- |
| Item | Discrimination | Difficulty | Discrimination | Difficulty |
| 26 | 0.90 | -0.25 | 49 | - |
| 27 | 1.36 | 1.90 | 52 | - |
| 30 | 1.87 | 1.17 | 75 | - |
| 32 | 1.72 | 1.45 | 117 | - |
| 49 | 1.11 | 2.88 | 204 | 2.40 |
| 50 | 1.36 | 0.67 | - | - |
| 52 | 1.17 | 0.27 | - | 0.33 |
| 62 | 1.37 | 1.24 | - | - |
| 75 | 2.05 | 1.41 | - | 1.21 |
| 78 | 0.92 | 1.50 | - | - |
| 117 | 2.07 | 1.89 | - | 2.12 |
| 129 | 1.54 | 1.81 | - | - |
| 152 | 1.53 | 0.83 | - | - |
| 161 | 1.45 | 1.26 | - | - |
| 204 | 1.42 | 0.83 | - | 0.99 |

Table S8. Item parameters for the Vulnerability To Harm schema

| Vulnerability To Harm | Original subscale | | 5-item subscale | |
| --- | --- | --- | --- | --- |
| Item | Discrimination | Difficulty | Discrimination | Difficulty |
| 56 | 1.73 | 1.05 | - | - |
| 71 | 1.35 | 2.35 | 1.43 | 2.27 |
| 98 | 1.58 | 1.16 | 1.76 | 1.09 |
| 109 | 1.35 | 2.03 | - | - |
| 110 | 1.36 | 1.72 | 1.32 | 1.74 |
| 122 | 0.76 | 1.84 | - | - |
| 133 | 1.72 | 1.09 | - | - |
| 134 | 1.20 | 0.59 | 0.96 | 0.66 |
| 146 | 0.72 | 1.21 | - | - |
| 149 | 1.54 | 0.70 | - | - |
| 174 | 1.96 | 1.34 | - | - |
| 178 | 1.01 | 0.77 | - | - |
| 197 | 1.72 | 1.42 | - | - |
| 198 | 1.98 | 1.44 | 1.93 | 1.43 |

Table S9. Item parameters for the Enmeshment schema

| Enmeshment | Original subscale | | 5-item subscale | |
| --- | --- | --- | --- | --- |
| Item | Discrimination | Difficulty | Discrimination | Difficulty |
| 86 | 0.99 | 2.50 | - | - |
| 99 | 1.08 | 1.76 | 0.93 | 1.96 |
| 125 | 1.75 | 1.40 | - | - |
| 126 | 1.83 | 1.21 | - | - |
| 138 | 1.40 | 1.71 | - | - |
| 143 | 2.00 | 1.33 | 2.28 | 1.26 |
| 144 | 1.68 | 1.24 | - | - |
| 154 | 1.49 | 2.28 | 1.24 | 2.57 |
| 162 | 1.96 | 1.34 | - | - |
| 183 | 2.50 | 1.17 | 2.32 | 1.20 |
| 188 | 2.38 | 1.23 | 2.98 | 1.16 |

Table S10. Item parameters for the Subjugation schema

| Subjugation | Original subscale | | 5-item subscale | |
| --- | --- | --- | --- | --- |
| Item | Discrimination | Difficulty | Discrimination | Difficulty |
| 39 | 2.23 | 0.90 | - | - |
| 40 | 1.25 | 0.64 | 1.10 | 0.67 |
| 72 | 2.66 | 1.16 | - | - |
| 83 | 1.57 | 1.44 | 1.46 | 1.49 |
| 94 | 1.19 | 1.12 | - | - |
| 100 | 1.55 | 1.15 | - | - |
| 113 | 1.50 | 0.31 | 1.59 | 0.29 |
| 142 | 2.59 | 0.93 | 3.19 | 0.88 |
| 159 | 1.82 | 1.30 | - | - |
| 193 | 1.06 | 1.85 | 0.92 | 2.04 |

Table S11. Item parameters for the Self-sacrifice schema

| Self-sacrifice | Original subscale | | 5-item subscale | |
| --- | --- | --- | --- | --- |
| Item | Discrimination | Difficulty | Discrimination | Difficulty |
| 22 | 0.64 | -0.13 | - | - |
| 34 | 1.22 | 0.11 | 1.35 | 0.14 |
| 63 | 0.96 | -0.01 | - | - |
| 68 | 1.13 | -0.54 | 0.96 | -0.62 |
| 76 | 0.97 | -0.53 | - | - |
| 112 | 1.37 | 0.04 | - | - |
| 136 | 0.82 | 0.90 | - | - |
| 140 | 1.49 | 0.31 | - | - |
| 141 | 1.85 | 0.60 | - | - |
| 156 | 1.23 | 0.29 | - | - |
| 160 | 1.85 | 0.96 | 1.93 | 0.97 |
| 185 | 1.48 | 0.14 | - | - |
| 187 | 0.88 | -0.51 | - | - |
| 189 | 1.84 | 0.85 | - | - |
| 196 | 2.25 | 0.42 | 2.62 | 0.43 |
| 199 | 1.12 | 1.47 | 1.34 | 1.32 |
| 202 | 1.24 | 0.29 | - | - |

Table S12. Item parameters for the Emotional Inhibition schema

| Emotional Inhibition | Original subscale | | 5-item subscale | |
| --- | --- | --- | --- | --- |
| Item | Discrimination | Difficulty | Discrimination | Difficulty |
| 6 | 1.39 | 1.07 | 1.12 | 1.22 |
| 36 | 1.29 | 1.45 | 1.33 | 1.42 |
| 151 | 1.53 | 1.12 | - | - |
| 165 | 2.21 | 1.14 | 2.80 | 1.07 |
| 170 | 1.47 | 1.52 | 2.02 | 1.30 |
| 179 | 1.73 | 1.15 | - | - |
| 190 | 1.92 | 1.10 | 2.04 | 1.09 |
| 195 | 1.56 | 1.14 | - | - |
| 201 | 1.99 | 1.12 | - | - |

Table S13. Item parameters for the Unrelenting Standards schema

| Unrelenting Standards | Original subscale | | 5-item subscale | |
| --- | --- | --- | --- | --- |
| Item | Discrimination | Difficulty | Discrimination | Difficulty |
| 17 | 1.57 | 0.66 | - | - |
| 25 | 1.52 | 1.03 | - | - |
| 55 | 1.41 | 1.32 | 1.40 | 1.32 |
| 65 | 1.22 | 1.63 | 1.46 | 1.47 |
| 79 | 0.92 | 1.27 | - | - |
| 118 | 1.16 | 0.18 | - | - |
| 131 | 2.23 | 0.82 | - | - |
| 147 | 1.22 | 0.09 | 1.04 | 0.09 |
| 153 | 1.49 | 0.42 | - | - |
| 157 | 1.21 | -0.09 | - | - |
| 168 | 1.36 | 1.06 | - | - |
| 171 | 1.05 | 0.85 | - | - |
| 180 | 1.54 | 1.43 | - | - |
| 186 | 0.66 | 2.36 | - | - |
| 203 | 2.25 | 0.33 | 2.03 | 0.33 |
| 205 | 2.04 | 0.72 | 1.69 | 0.79 |

Table S14. Item parameters for the Entitlement schema

| Entitlement | Original subscale | | 5-item subscale | |
| --- | --- | --- | --- | --- |
| Item | Discrimination | Difficulty | Discrimination | Difficulty |
| 21 | 1.48 | 0.91 | - | - |
| 48 | 0.84 | 1.90 | - | - |
| 91 | 1.61 | 1.44 | - | - |
| 93 | 1.76 | 1.52 | 1.96 | 1.45 |
| 102 | 1.19 | 0.20 | 1.24 | 0.19 |
| 105 | 1.57 | 1.69 | 1.06 | 2.19 |
| 111 | 1.04 | -0.14 | 1.15 | -0.12 |
| 139 | 1.24 | 1.51 | - | - |
| 182 | 1.41 | 1.52 | - | - |
| 191 | 2.27 | 0.81 | 2.46 | 0.79 |
| 200 | 1.17 | 1.48 | - | - |

Table S15. Item parameters for the Insufficient Self-control schema

| Insufficient Self-control | Original subscale | | 5-item subscale | |
| --- | --- | --- | --- | --- |
| Item | Discrimination | Difficulty | Discrimination | Difficulty |
| 10 | 0.62 | 2.61 | - | - |
| 13 | 1.29 | 0.64 | - | - |
| 46 | 2.20 | 1.11 | 46 | 1.61 |
| 51 | 1.00 | 1.76 | 51 | 1.22 |
| 59 | 1.08 | 1.00 | - | - |
| 67 | 1.11 | 1.04 | - | - |
| 73 | 1.09 | 0.65 | 73 | 0.59 |
| 84 | 1.20 | 1.00 | - | - |
| 85 | 2.02 | 1.00 | 85 | 1.34 |
| 88 | 1.74 | 0.92 | - | - |
| 92 | 1.22 | 1.94 | 92 | 1.53 |
| 108 | 2.10 | 0.87 | - | - |

**YSQ-GeMS Item List (Dutch Version)**

Table S16. Overview of YSQ-GeMS items in Dutch, showing the preserved original item order, newly assigned item numbers, and corresponding item numbers from the original YSQ-L2

| YSQ-GeMS number | Original YSQL2 number |  |
| --- | --- | --- |
| 1 | 2 | Wanneer ik merk dat iemand om wie ik geef, afstand van me neemt, word ik wanhopig. |
| 2 | 3 | Ik ben een mislukkeling. |
| 3 | 4 | Zodra het op presteren aankomt, ben ik tot weinig in staat. |
| 4 | 6 | Ik schaam me ervoor om mijn gevoelens naar anderen te uiten. |
| 5 | 7 | Ik voel me vervreemd van andere mensen. |
| 6 | 9 | Het ligt aan mij dat mijn ouders niet genoeg van mij hebben kunnen houden. |
| 7 | 11 | Als iemand aardig tegen mij doet, neem ik aan dat hij of zij ergens op uit is. |
| 8 | 18 | Ik raak van slag als iemand me alleen laat, ook al is het maar voor even. |
| 9 | 24 | Als ik het idee heb dat iemand erop uit is om mij te kwetsen, probeer ik diegene als eerste te kwetsen. |
| 10 | 31 | Ik pas er niet bij. |
| 11 | 34 | Ik voel me schuldig als ik andere mensen teleurstel. |
| 12 | 36 | Ik voel steeds meer woede en haat, die ik opkrop. |
| 13 | 40 | Ik doe veel meer moeite dan de meeste anderen om conflicten uit de weg te gaan. |
| 14 | 42 | Ik maak me zorgen dat de mensen die me dierbaar zijn, me in de steek zullen laten. |
| 15 | 44 | Ik heb nooit liefde en aandacht gekregen. |
| 16 | 46 | Ik heb me bijna nooit aan mijn voornemens kunnen houden. |
| 17 | 47 | Ik voel dat ik iemand ben waar niemand van kan houden. |
| 18 | 49 | Ik verpest alles wat ik doe, zelfs buiten mijn werk (of opleiding). |
| 19 | 51 | Het gebeurt vaak dat als ik eenmaal kwaad word, ik mezelf niet in de hand kan houden. |
| 20 | 52 | Ik heb andere mensen nodig om me ergens doorheen te slaan. |
| 21 | 54 | Bijna niets wat ik doe in mijn werk (of studie) haalt het bij wat andere mensen kunnen. |
| 22 | 55 | Ik moet de beste zijn in bijna alles wat ik doe; ik accepteer geen tweede plaats. |
| 23 | 60 | Andere mensen zijn gewoonlijk niet wat ze lijken te zijn; ze zijn bijna nooit eerlijk. |
| 24 | 64 | Ik voel me alleen en geïsoleerd van andere mensen. |
| 25 | 65 | Ik ben iemand die altijd concurreert met anderen. |
| 26 | 68 | Ik ben altijd degene die luistert naar de problemen van anderen. |
| 27 | 71 | Ik ben bang dat ik dakloos raak of zwerver word. |
| 28 | 72 | Als ik niet toegeef aan de wensen van anderen, pakken ze me op de één of andere manier terug of wijzen ze me af. |
| 29 | 73 | Ik heb de neiging te hard van stapel te lopen, ook al weet ik dat dat slecht voor me is. |
| 30 | 75 | Mijn oordeel in alledaagse situaties is niet betrouwbaar. |
| 31 | 82 | Een van mijn grootste angsten is dat mijn tekortkomingen aan het licht komen. |
| 32 | 85 | Als taken moeilijk worden, kan ik het meestal niet opbrengen om ze af te maken. |
| 33 | 89 | Ik ben de liefde, de aandacht en het respect van anderen niet waard. |
| 34 | 92 | Bij de kleinste op- of aanmerking word ik al boos. |
| 35 | 93 | Ik word vaak geïrriteerd of boos als ik niet krijg wat ik wil. |
| 36 | 96 | Er is bijna nooit iemand geweest bij wie ik terecht kon voor advies en emotionele steun. |
| 37 | 98 | Ik kan maar niet aan het gevoel ontkomen dat er iets ergs staat te gebeuren. |
| 38 | 99 | Ik ben niet in staat geweest me los te maken van mijn ouder(s) zoals anderen van mijn leeftijd dat wel lijken te hebben gedaan. |
| 39 | 101 | Soms ben ik zo bang dat mensen me verlaten, dat ik ze juist wegjaag. |
| 40 | 102 | Ik kan er niet tegen dat andere mensen me zeggen wat ik moet doen. |
| 41 | 103 | Ik voel me soms volledig een buitenstaander. |
| 42 | 105 | Ik vind dat ik niet hoef te voldoen aan de normale regels en afspraken. |
| 43 | 107 | Ik vergelijk hetgeen ik heb bereikt vaak met wat anderen hebben bereikt en vind dat zij veel succesvoller zijn. |
| 44 | 110 | Ik ben bang dat ik een ernstige ziekte onder de leden heb, ook al is er door de dokter niets ernstigs geconstateerd. |
| 45 | 111 | Ik vind het heel vervelend als ik beperkt of verhinderd word om te doen wat ik wil. |
| 46 | 113 | Ik vind het erg moeilijk om te eisen dat mijn rechten worden gerespecteerd en dat er rekening wordt gehouden met mijn gevoelens. |
| 47 | 117 | Het ontbreekt mij aan gezond verstand. |
| 48 | 120 | Als ik morgen zou verdwijnen, zou niemand het merken. |
| 49 | 121 | Er zijn in mijn leven maar weinig mensen geweest die echt naar mij luisterden, me begrepen of die oog hadden voor mijn ware behoeften en gevoelens. |
| 50 | 127 | De meeste mensen denken alleen aan zichzelf. |
| 51 | 130 | Ik denk dat mensen misbruik van me zullen maken. |
| 52 | 132 | Ik voel me aangetrokken tot partners die zich niet aan mij willen binden. |
| 53 | 134 | Ik pieker vaak over de slechte dingen die in de wereld gebeuren: criminaliteit, milieuvervuiling, enzovoorts. |
| 54 | 137 | Ik ben van nature slecht en onvolmaakt. |
| 55 | 142 | Ik geef andere mensen hun zin, omdat ik bang ben voor de gevolgen als ik dat niet zou doen. |
| 56 | 143 | Ik ben zo betrokken bij mijn partner of ouder(s), dat ik niet meer weet wie ik zelf ben of wat ik zelf wil. |
| 57 | 147 | Ik streef ernaar dat bijna alles perfect in orde is. |
| 58 | 154 | Mijn ouder(s) en ik moeten bijna elke dag met elkaar praten, anders voelt één van ons zich schuldig, gekwetst, teleurgesteld of alleen. |
| 59 | 155 | Ik heb zelden het gevoel gehad dat ik bijzonder ben voor iemand. |
| 60 | 160 | Ik ben zo druk met anderen om wie ik geef, dat ik weinig tijd voor mijzelf overhoud. |
| 61 | 165 | Ik moet mijn emoties en impulsen beheersen, want anders gebeurt er waarschijnlijk iets ergs. |
| 62 | 166 | Ik schaam me vaak als ik bij anderen ben, omdat zij veel meer bereikt hebben dan ik. |
| 63 | 170 | Ik ben bang anderen ernstig te beschadigen (lichamelijk of emotioneel), als ik de beheersing over mijn boosheid verlies. |
| 64 | 183 | Ik heb vaak het idee dat ik geen privacy heb bij mijn ouder(s) of partner. |
| 65 | 184 | Meestal was er niemand die voor me zorgde, dingen met me deelde of die het echt kon schelen wat er met me gebeurde. |
| 66 | 188 | Ik heb vaak het idee dat ik geen eigen identiteit heb ten opzichte van mijn ouder(s) of partner. |
| 67 | 190 | Ik ben bang de controle over mijn gedrag te verliezen. |
| 68 | 191 | Ik heb grote moeite een weigering te accepteren als ik iets van andere mensen wil. |
| 69 | 193 | Ik pak mensen terug op kleine dingen in plaats van mijn boosheid te tonen. |
| 70 | 196 | Ik geef voorrang aan de behoeften van andere mensen boven die van mijzelf, anders voel ik me schuldig. |
| 71 | 198 | Ik heb vaak het idee dat ik gek zou kunnen worden. |
| 72 | 199 | Als ik doe wat ik wil, voel ik me niet op mijn gemak. |
| 73 | 203 | Bijna niets wat ik doe is echt goed genoeg; het zou altijd beter kunnen. |
| 74 | 204 | Ik voel mij niet in staat me alleen te redden in het dagelijks leven. |
| 75 | 205 | Ik zet mijzelf zo onder druk om het goed te doen, dat dit ten koste gaat van mijn gezondheid. |
